# Evaluating Antimicrobial Effectiveness of Gold Nanoparticles against *Streptococcus oralis*

**DOI:** 10.1155/2023/9935556

**Published:** 2023-09-20

**Authors:** Batool M Al-Fahham, Rafeef Ahmed Mohamed, Juman Mohammed Taqi Al-Talqani, Ali Hadi Fahad, Julfikar Haider

**Affiliations:** ^1^Department of Pediatric and Preventive dentistry, College of Dentistry, University of Kufa, Iraq; ^2^Department of Engineering, Manchester Metropolitan University, Manchester, M12 5GN, UK

## Abstract

Biofilm includes many microorganisms that causes the periodontal diseases. The increased drugs resistance against the infectious diseases is a major issue owing to excessive using of a broad spectrum of antibiotics. Recently, metallic nanoparticles (NPs) are being administered to control the growth of different types of microorganisms. For instance, gold nanoparticles (Au NPs) are found to be successful to control and limit the bacterial pathogenicity in the oral cavity without any cytotoxic effects on the human body. *Aim*. In this paper, it was aimed to detect the antibacterial effect of Au NPs and compare with chlorhexidine (CHX) against *Streptococcus oralis* (S. *oralis*) in dental plaque of patients with chronic periodontitis. *Materials and Methods*. First, supragingival and subgingival plaque samples were collected from the patients suffering from periodontal disease and incubated under aerobic or/and anaerobic conditions. Second, the morphological examination, and biochemical test by Vitec 2 machine are used to confirm the *S*. *oralis* species. Third, the synthesis of Au NPs was carried out by seed growth method and their properties were characterized. Finally, the antimicrobial effect of the Au NPs against *S. oralis* was evaluated by Agar well diffusion method for different Au NPs concentrations (100, 50, 25, 12.5, 6.25, 3.125, 1.562, 0.781, 0.391, 0.195, and 0.097 ppm). CHX was used as the positive control and distilled water as the negative control. The antibacterial activity data were statistically analyzed by least significant difference (LSD) using the Statistical Program for Social Science (SPSS) version 22. *Results*. The Au NPs with an average particles size of 43 nm, polycrystalline face-centered cubic structure were characterized. The Au NPs at 100 ppm concentration had similar antibacterial effect of CHX for inhibiting the growth of S. *oralis*, with no significant difference. *Conclusions*. The Au NPs as an antibacterial agent could be equally effective against *S. oralis* similar to the CHX when used at higher concentration.

## 1. Introduction

Periodontal diseases are inflammatory and damaging and are generally resulted from periodontal pathogens residing in periodontal pockets. Multimicrobial dental biofilm can cause periodontal diseases and dental caries in the oral cavity [1]. Various types of microorganisms are present in the extracellular matrix of the biofilm [2] forming a multicellular entity. Biofilm removal is highly challenging when it is present between the gingival sulcus and teeth. The matrix of excreted polymeric substances (EPS) holding the biofilm complex together, providing nutrients, adhesion, protection, and stabilization in the biofilm [3].

The immersion of the tooth surfaces in the oral cavity environment after cleaning the tooth results in surface assimilation of a thin layer of acquired pellicle. The pellicle consists of glycoproteins of saliva, for example statherin, proline-rich proteins, agglutinin, a-amylase, and mucins [4].

The bacteria are attached on the tooth surfaces in different manners, ranging from strong interaction between molecules of bacterial surface and components of pellicle to weak charge-mediated interactions [5–7]. The Gram-positive stain and facultative anaerobic with different shape such as cocci and rods bacteria that initially colonized the teeth. The most common species are Streptococcus and Actinomyces [8].

The oral streptococci are a commensals bacterium that normally inhabit the gastrointestinal and genitourinary tracts, in addition to the teeth surfaces and oral mucosa. In healthy individuals, Streptococci may form more than 50% of the oral microbiota. The family of Streptococcaceae is classified under the Firmicutes phylum and Lactobacillales order [8, 9] and have three important genus: Lactococcus, Streptococcus, and Lactovum. Among them the Streptococcus is more diverse having 79 species. The Streptococcus having species which are considered as pathogenic in humans and animals for instance *S. pneumonia* and *S. pyogenes* are the significant bacterial pathogens [8]. Generally, the Gram-positive bacteria showed pairs or/and chains with spherical shape colonies and many of the Gram-positive bacteria form capsules [10].

According to the U.S. Centers for Disease Control and Prevention, the bacteria resistant to antibiotic drug led to an increase of infections and deaths in the United States causing a pressing need for developing new effective antibiotics and treatment approaches [11]. NPs are considered as the important materials to develop new devices in a number of applications including biomedical, biological, and pharmaceutical [12, 13]. Among the NPs, gold NPs (Au NPs) are profoundly used as an agent in the medical therapy, diagnostic, and gene therapy [14, 15]. The major advantage of the Au NPs which are synthesized simply by a technique called chemical reduction is reduced toxicity in contrast to other nanomaterials. The results of synthesized Au NPs demonstrated a higher and moderate level of antibacterial activity against Gram-negative and Gram-positive bacteria, respectively [16]. It should be noted that the gold ions released from the Au NPs is useful in creating the antibacterial activity [17, 18].

So far, no study has been carried out on evaluating the antibacterial effect of the Au NPs against *S. oralis* collected from patients suffering from periodontal diseases. The aim of this research is to synthesize and characterize the Au NPs and assess their effectiveness against *S. oralis*. The null hypothesis states that Au NPs are not effective against *S. oralis* at different concentrations from 0.097 to 100 ppm.

## 2. Materials and Methods

**Figure 1:**
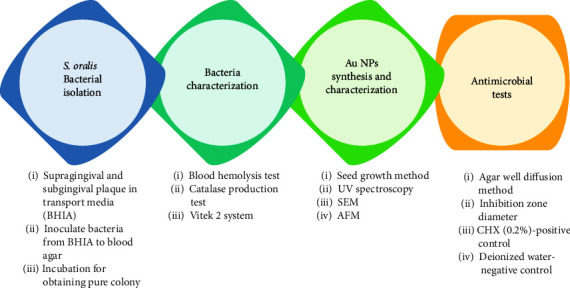
Workflow for evaluating antimicrobial effectiveness of Au NPs against *S. oralis*.

The workflow of this investigation started with isolation of *S. oralis* bacteria followed by microscopical observations and biochemical tests to confirm the identity of the bacteria (Figure 1). The next steps included synthesis of Au NPs and characterize their features. Finally, the potential antimicrobial by Au NPs on *S. oralis* in contrast to 0.2% CHX (positive control) and deionized water (negative control).

### 2.1. Bacterial Isolation and Staining

**Figure 2:**
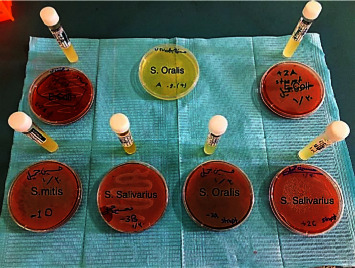
Bacterial growth in BHIA and blood agar.

The plaque samples from 10 patients were gathered. Two samples from each patient with periodontal disease (subgingival pocket at a depth of 4–5 mm) and incubated both aerobically and anaerobically. The sample size was selected in such a way to increase the chance of obtaining the desired bacteria. The following exclusion criteria were considered during the sample collection. For instance, the teeth were caries-free; patients did not take any antibiotic 3 month prior to the sample collection and patients did not undergo any periodontal treatment within the last 3 months including scaling and subgingival application of any antiplaque agents. The reason for collecting the samples from two areas just to further increase the chance of obtaining the right bacteria. Relevant consents were acquired from each patient informing that the samples will be used for conducting academic research. All samples were placed in a transport media containing brain heart infusion agar (BHIA) and incubated for 24 hr at 37°C in an aerobic incubator from (BINDER, Germany) for supragingival samples and in a CO_2_ incubator (New Brunswick, Germany) for subgingival samples, and inoculated the bacteria from BHIA to blood agar. Subsequently, the samples were incubated over night at 37°C in two conditions, aerobic and CO_2_ incubators to distinguish it from other bacteria. The bacterial subculture was continued to get a pure colony (Figure 2) [19].

After picking up the colony from the blood agar, the Gram staining was performed according to Thairu et al. [2,0]. The single colony was placed on a glass slide and mixed with a drop of pure water, then, dried and fixed by heating followed by crystal violet stain for 1 min, then washed by distilled water carefully. Lugols iodine was applied for 1 min then washed by acetone for 30 s to remove the color. The last step included safranin staining for 1 min then washing and drying. A light microscope (Feica, Germany) with a magnification of ×100 was used for slide examination under oil immersion.

### 2.2. Biochemical Tests

**Figure 3:**
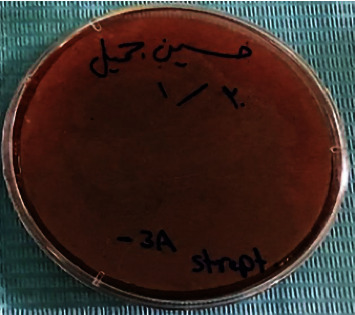
*α*-hemolysis of *S. oralis* on blood agar.

To evaluate the capability of S. *oralis* in oxidizing the hemoglobin to methemoglobin by hydrogen peroxide (H_2_O_2_), the blood hemolysis test was done. The *S. oralis* showed *α*-hemolysis and a greenish color on blood agar was observed resulted from bleaching of heme iron, as illustrated in Figure 3.

Catalase test was carried out to detect catalase enzyme using hydrogen peroxide (3% H_2_O_2_) [21] for the purpose of identifying bacterium types. A Vitek 2 system (VK2C9513, USA) was used to verify the specific bacterial type [22]. The system results detected the presence of *S. oralis* with 90% probability.

### 2.3. Synthesis and Characterization of Au NPs

**Figure 4:**
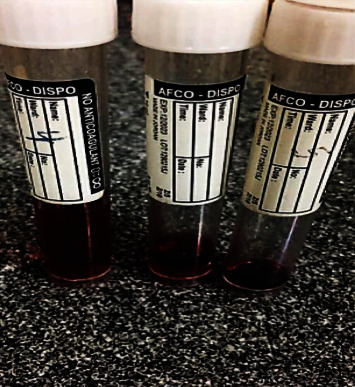
Gold NPs suspension.

The Au NPs synthesis method involved preparation of a seed solution and a growth solution [23] separately followed by mixing these two solutions to get one Au NPs suspension. Distilled water was used as a medium for preparing both the solutions. Chloroauric acid, ascorbic acid, sodium borohydride, cetyl trimethylammonium bromide (CTAB), and trisodium citrate were used for Au NPs synthesis. The resultant suspension displayed a red color which indicated the synthesis of the Au NPs (Figure 4).

Characterizations of the Au NPs were carried out using an ultraviolet (UV) visible spectroscope (Shimadzu, Japan), an X-ray diffractometer (XRD, Bruker, Germany), an electron microscope (SEM, FEI, Netherlands), and microscope of Atomic Force (AFM, Bruker, Germany) [24].

### 2.4. Agar Well Diffusion Test

The antibacterial effect of the Au NPs on *S. oralis*, agar well diffusion processor was used [25]. Sterile petri dishes were used to prepare agar media and left overnight to become set. The bacterial inoculums were produced by adding 0.1 ml of bacterial culture to 10 ml of BHIA and then incubated aerobically for 24 hr at 37°C to allow the bacterial growth. 0.1 ml of activated *S. oralis* suspension at 0.5 McFarland tube were spread twice on Mueller Hinton Agar (MHA) agar plates and inoculated the indicated bacteria on agar by leaving at room temperature for 10 min. Wells of equal size and depth (4 mm in diameter) were punched in the MHA agar with a clean stainless steel cork borer. Each well was filled with Au NPs (25 *µ*L) at different concentrations (100, 50, 25, 12.5, 6.25, 3.125, 1.562, 0.781, 0.391, 0.195, and 0.097 ppm). Positive and negative controls were prepared by filling one well with 0.2% CHX and another well by deionized water. The tests were repeated in three petri dishes (*n* = 3). The plates were left for 10 min at the room temperature and then incubated aerobically for overnight at 37°C. To determine the minimum inhibitory concentration (MIC), the diameter of inhibition zones was measured and recorded after the incubation.

### 2.5. Statistical Analysis

The data were statistically analyzed and significance at *P* < 0.05 was determined using SPSS software (Statistical for Social Science, Version 22, IBM Corp., USA). The descriptive statistics including mean and standard deviation and least significant difference (LSD) were used to evaluate antibacterial effect of the Au NPs against the *S. oralis* bacteria.

## 3. Results

### 3.1. Morphological Properties of S. oralis

**Figure 5:**
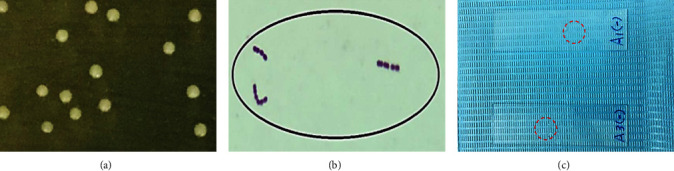
Characteristics of *S. oralis* colony (a) in BHIA, (b) after Gram stain (magnification ×100), and (c) after catalase test (absence of bubbles).

*S. oralis* was found in supragingival and subgingival areas and no difference in morphological characteristics between them was found. *S. oralis* bacteria appeared small, round, smooth, glistening, and convex colonies, as shown in Figure 5(a). After staining with Gram stain, *S. oralis* bacteria appeared on microscope as Gram positive, cocci shape, arranged in long and short chains (Figure 5(b)).  Figure 5(c) showed evidence that *S. oralis* was catalase negative since no catalase enzyme was produced, which was evidenced by the absence of oxygen gas bubbles.

### 3.2. Characteristics of Synthesized Au NPs

**Figure 6:**
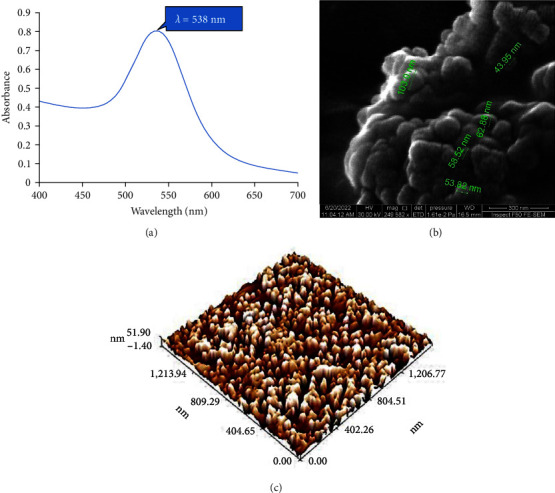
(a) Absorption spectra; (b) FE-SEM micrograph; (c) 2D; and (d) 3D AFM image of Au NPs.

Optical and structural properties of the prepared NPs was evaluated by the UV-visible absorption spectroscopy.  Figure 6(a) showed the absorption band characteristics of the Au NPs where the plasmon peak was found at a wavelength around 538 nm, which was associated with the surface plasmon resonance (SPR). The spectra showed a single SPR and this was a strong indication for uniform dispersion of the Au NPs in the suspension.

The full emission SEM image showed spherical shape and homogenous well dispersing of Au NPs (Figure 6(b)). The AFM images of the Au NPs synthesized by the seeding growth method showed that the average grain size was 43.36 nm and they were evenly distributed (Figure 6(c)).

The crystal structure of the Au NPs established by XRD revealed sharp diffraction peaks at 12.3°, 21.8°, and 51.8°, which represented the crystal planes of (110), (200), and (220), respectively. Therefore, it can be confirmed that the Au NPs are polycrystalline in nature with face-centered cubic structure.

### 3.3. Results of Agar Well Diffusion Method

**Figure 7:**
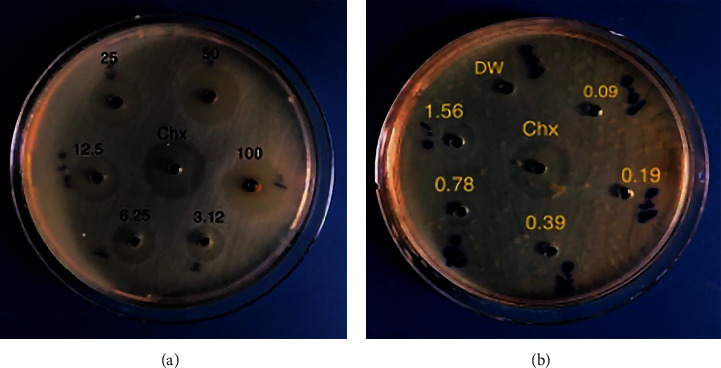
Inhibition zones created by Au NPs against *S. oralis* for concentrations of (a) 3.12, 6.25, 12.5, 25, 50, and 100 ppm; and (b) 0.09, 0.19, 0.39, 0.78, and 1.56 ppm.

**Figure 8:**
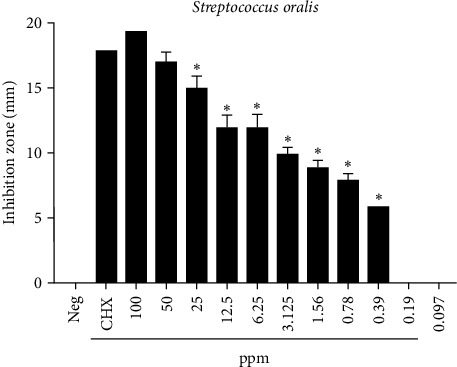
The antimicrobial activity of Au NPs against *S. oralis* ^*∗*^*P* < 0.05.

The well diffusion results showed that the highest antibacterial activity was achieved when an Au NPs concentration of 100 ppm was used against *S. oralis* as the diameter of zone of inhibition was 19.5 mm. While the inhibition zone decreased to 6 mm for an Au NPs concentration of 0.39 ppm and this indicated an MIC. Moreover, this concentration was recorded significantly different in antibacterial activity (*P* < 0.05) from that by CHX, as shown in Figure 7. No inhibition zone was found for concentrations less than 0.39 ppm. On the other hand, the result of CHX as the positive control showed 18 mm inhibition zone diameter and deionized water as the negative control displayed no antibacterial activity (Table 1 and Figure 8).

## 4. Discussion

The uprising of the infectious diseases induced by a variety of bacterial pathogens and development of their resistance against different drugs motivated the scientific researchers in seeking advance antibacterial agents. Nanomaterials have been developed to inhibit bacterial pathogenicity, which possess special chemical and physical features [26, 27].

The Au NPs were selected in this study due to its proven high antimicrobial activity [28]. The Au NPs are attached to the membrane of the bacterium by interacting electrostatically, which subsequently damages its structure [29]. They lower the levels of adenosine triphosphate (ATP) in the cell, changing the membrane potential, inhibiting the binding of tRNA in the ribosomal, and influence the translation process [30]. In addition, the Au NPs showed no toxicity, therefore it can be used as a biocompatible agent, and have been incorporated into a variety of biomedical systems and proved to be an effective antimicrobial agent [31].

Oral Streptococci of the Mitis group [32, 33] such as *S. oralis*, is associated with the *S. pneumonia* which is considered to be the important bacteria pathogen and it is the early colonizer in the development of oral biofilms [7]. In the present study, bacterial samples from supragingival area and subgingival pocket (4–5 mm pocket depth) were taken from 10 patients who fulfill the inclusion criteria to isolate the *S. oralis*. The agar well diffusion method was applied to assess the antibacterial activation and determine an MIC of the Au NPs against the *S. oralis*. Then, the antimicrobial impacts of the Au NPs at various concentrations were compared with the 0.2% CHX, a positive control and the deionized water, and a negative control against the specific bacteria. The agar well diffusion process was performed in this study due to its simplicity, reliability, not requiring any expensive equipment, and its wide acceptance as a gold standard technique [34].

Chlorhexidine (CHX) is the most common agent used as the chemical method for local plaque control. It has been reported that CHX is an active agent in the mouthwash. Furthermore, it has been mentioned that CHX is an important antiseptic with good positive results [35]. In addition to that, CHX affected a wide range of Gram-positive and Gram-negative bacteria and fungi [36]. The previous findings have shown that by administering 10 ml of CHX gluconate (0.2%) twice daily, it may work in inhibiting bacterial dental plaque. Moreover, many studies confirmed that gingivitis and periodontitis disease may be prevented by CHX [37, 38]. Clinical investigations using CHX for a long time as a mouthwash recorded a 45%–61% decrease in the level of the microbial plaque, while at the same time, a 27%–67% decrease in gingivitis disease [37]. However, despite of its positive points, numerous negative effects have been recorded most often correlated to its topical or oral employment as it is associated with reversible discoloration (tongue, teeth, and silicate or composite restorations). Therefore, it must not be used with dentifrices simultaneously, due to the interaction of CHX with fluoride and detergents present in the toothpaste. The products of CHX must be used 30 min after brushing. Moreover, a short-term burning sensation and a disturbing taste can be felt on the initial employment. Also, with the using of CHX mouthwash, oral desquamation, and unusual swelling of the parotid gland have been documented [39]. Therefore, this research is seeking an alternative antibacterial agent to substitute CHX.

The results of this study showed that both the Au NPs and CHX had an excellent antimicrobial activity against *S. oralis* compared to the deionized water as indicated by the inhibition zone diameters (*P* < 0.05). The Au NPs at 100 ppm and 50 ppm concentrations were equally effective same as the CHX against *S. oralis* and there was no significance difference between CHX and Au NPs at 50 ppm and 100 ppm, while there was a significance difference between them at low concentrations (12.5, 6.25, 3.125, 1.562, 0.781, and 0.391 ppm) of the Au NPs.

The current results agreed with the investigation conducted by Xiaoning et al. [40] who established that Au NPs could be used as antibacterial agent against multidrug-resistant Gram-positive and Gram-negative bacterial pathogens due to its favorable surface chemistry. Senthilkumar et al. [41] demonstrated that antibacterial activity of the Au NPs in a variety of concentration levels could inhibit Gram-positive bacteria and Gram-negative bacteria. Maysa et al. [42] revealed that Au NPs at high-level concentrations (75, 100, and 200 *µ*g/ml) had an impact on the biofilm formation in comparison to its low concentration. Behbahan et al. [43] determined that an MIC of the Au NPs at 100 ppm concentration, slowed down the growth of six strains from *Salmonella spp*. Some studies investigated antibacterial activity of the Au NPs synthesized from the extracts of *Ocimum tenuiflorum* flowers and leaves, *Azadirachta indica* and *Mentha spicata* leaves and *Citrus sinensis* peels [44]. An Au NPs concentration of 512 *µ*g/ml was recorded to highly active against S. *aureus*, *P. aeruginosa*, and *K. pneumoniae*. It was also reported that Au NPs extracted from the *Annona muricata* leaves could show antibacterial activity against two types of bacteria (Gram-positive and Gram-negative, for example, *E. faecalis*, *C. sporogenes*, *S. aureus*, and *K. pneumoniae*). In addition, Au NPs extracted from *Azadirachta indica* leaf and *Zingiber officinale* root could inhibit the progression of *S. mutant*, *E. faecalis*, and *S. aureus* bacteria [45, 46].

More recently, other compounds have shown a significant impact on the oral cavity such as probiotics [47], postbiotics [48], and lysates [49] that can modify clinical and microbiological indicators in periodontal patients. Therefore, these products should be investigated in future clinical studies especially combined with the Au NPs.

However, the major limitations of using Au NPs are considered to be its cost and difficulty in preparation. Dental biofilms contain hundreds of species of bacteria. It is very challenging to include all types of bacteria responsible of periodontal disease in one study. However, *S. oralis* is the primary periodontal colonizer; hence, this study was focused on this particular species. Further studies will be carried out with other microorganisms to realize the wider applicability of Au NPs as an antimicrobial agent.

## 5. Conclusion

Au NPs at different concentrations were tested as an antimicrobial agent against *S. oralis* collected from the patients with periodontal disease. The Au NPs at 100 ppm concentrations were equally effective as CHX against *S. oralis* making it as a suitable alternative to the commercial antibiotics.

Further in vitro and in vivo investigations should be carried out to confirm the functionality of the Au NPs as the antimicrobial agent against colony forming microorganisms in the field of dentistry.

## Figures and Tables

**Table 1:** Descriptive statistics of antibacterial activity at the different concentrations of Au NPs, CHX, and deionized water used in the study against *S. oralis* using well diffusion method.

Concentrations	*N*	Mean (mm)	SD (mm)	Minimum (mm)	Maximum (mm)
100	3	19.5	0.25	19.5	19.5
50	3	17	1	16	18
25	3	15	1	14	16
12.5	3	12	1	11	13
6.25	3	12	1	11	13
3.125	3	10	0.5	9.5	10.5
1.56	3	9	0.5	8.5	9.5
0.78	3	8	0.5	7.5	8.5
0.39 (MIC)	3	6	0.25	6	6
0.19	3	0	0	0	0
0.097	3	0	0	0	0
CHX 0.2%	3	18	0.25	18	18
D.W	3	0	0	0	0

D.W, distilled water.

## Data Availability

The data used to support the findings of this study are included within the article.
